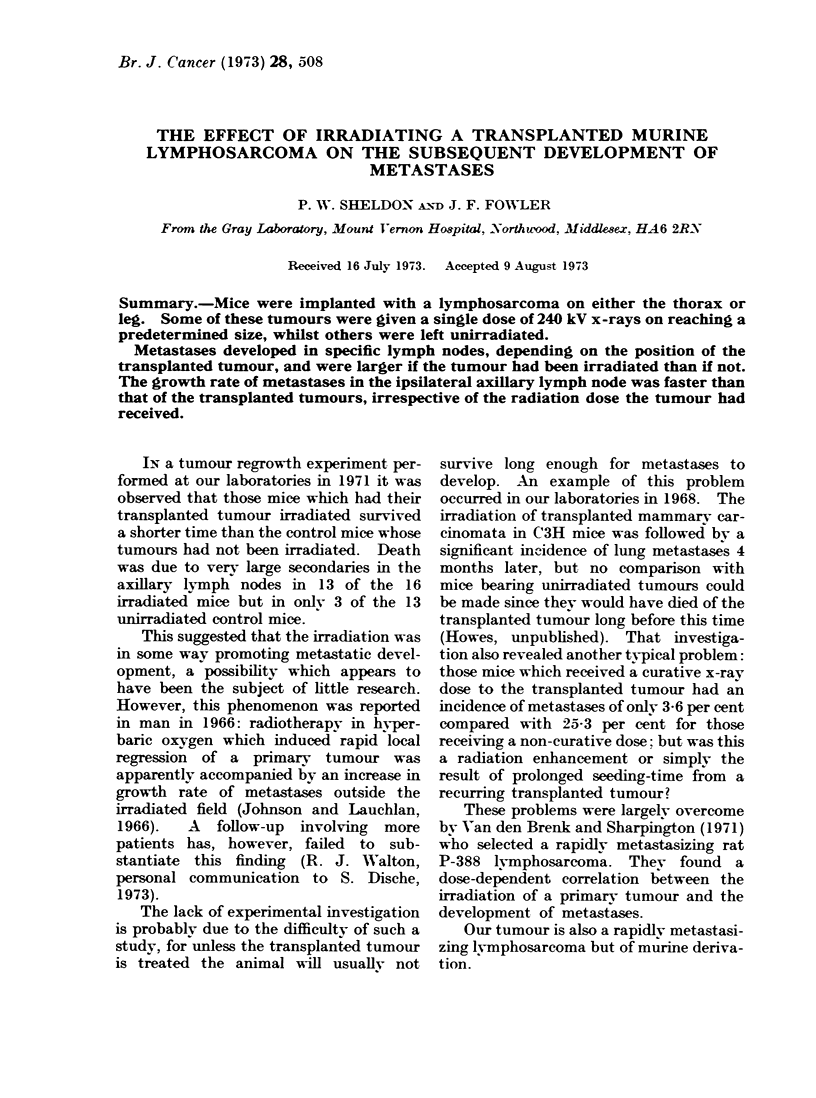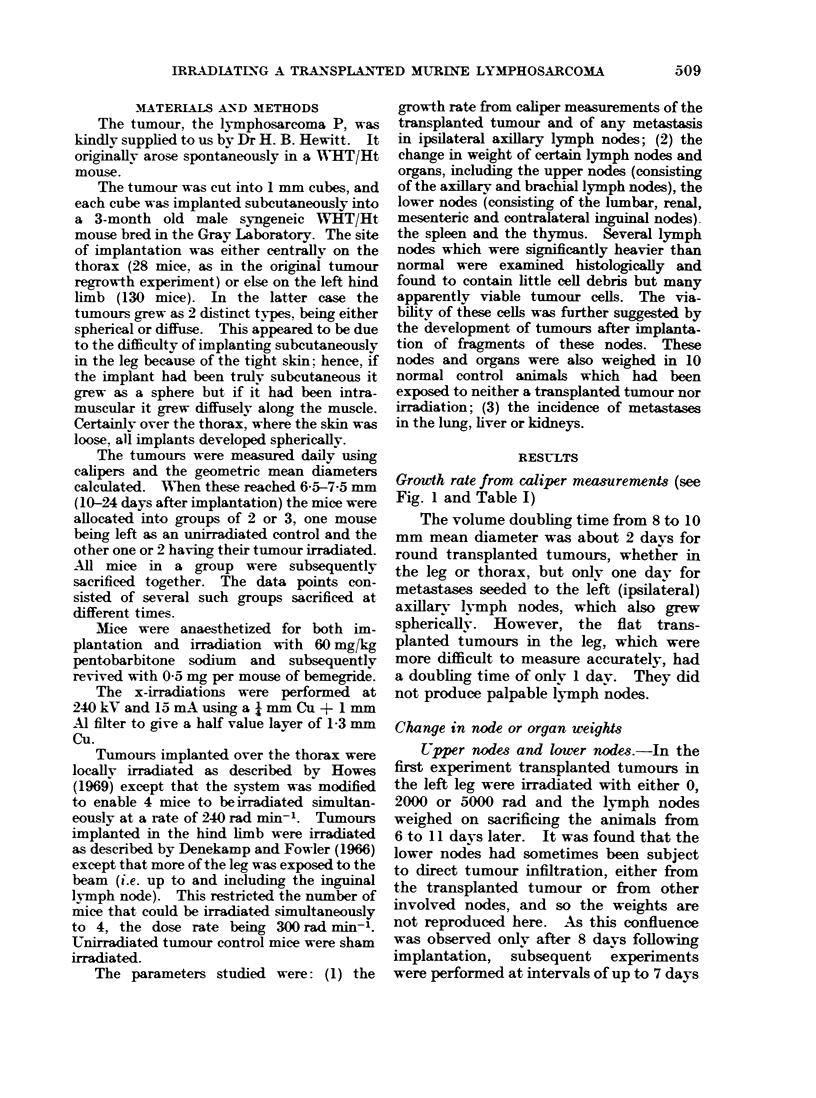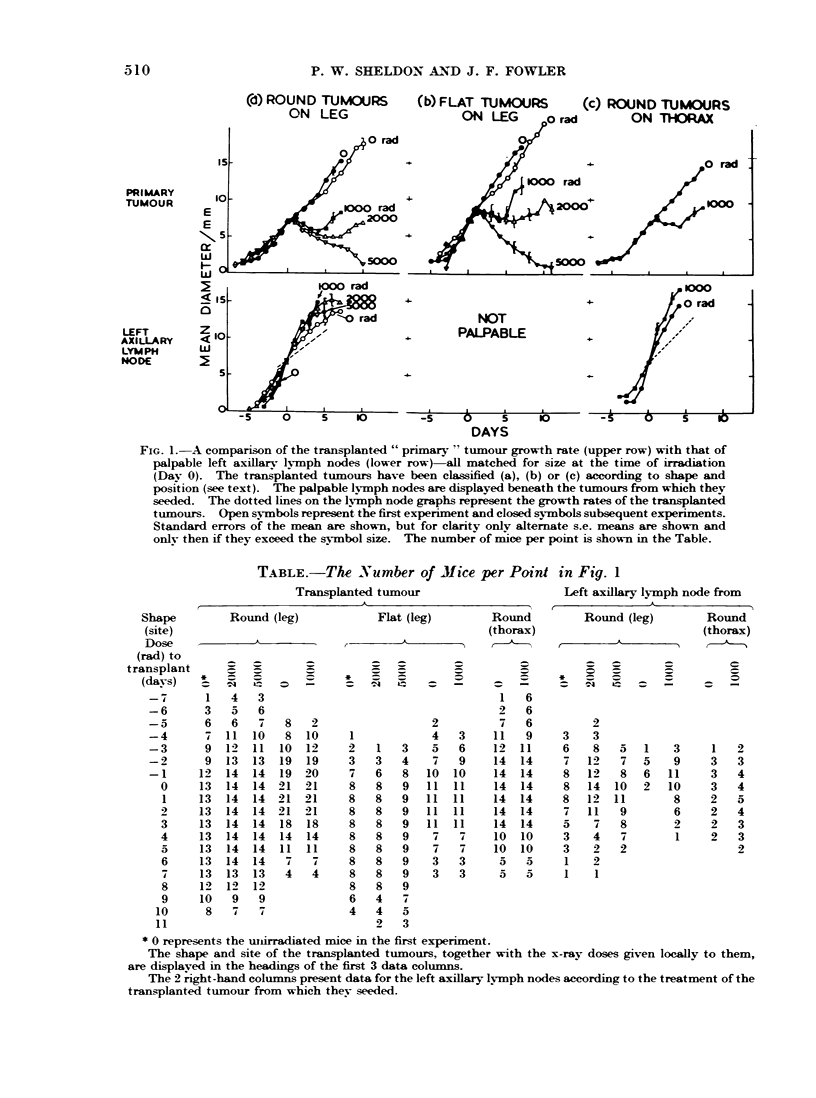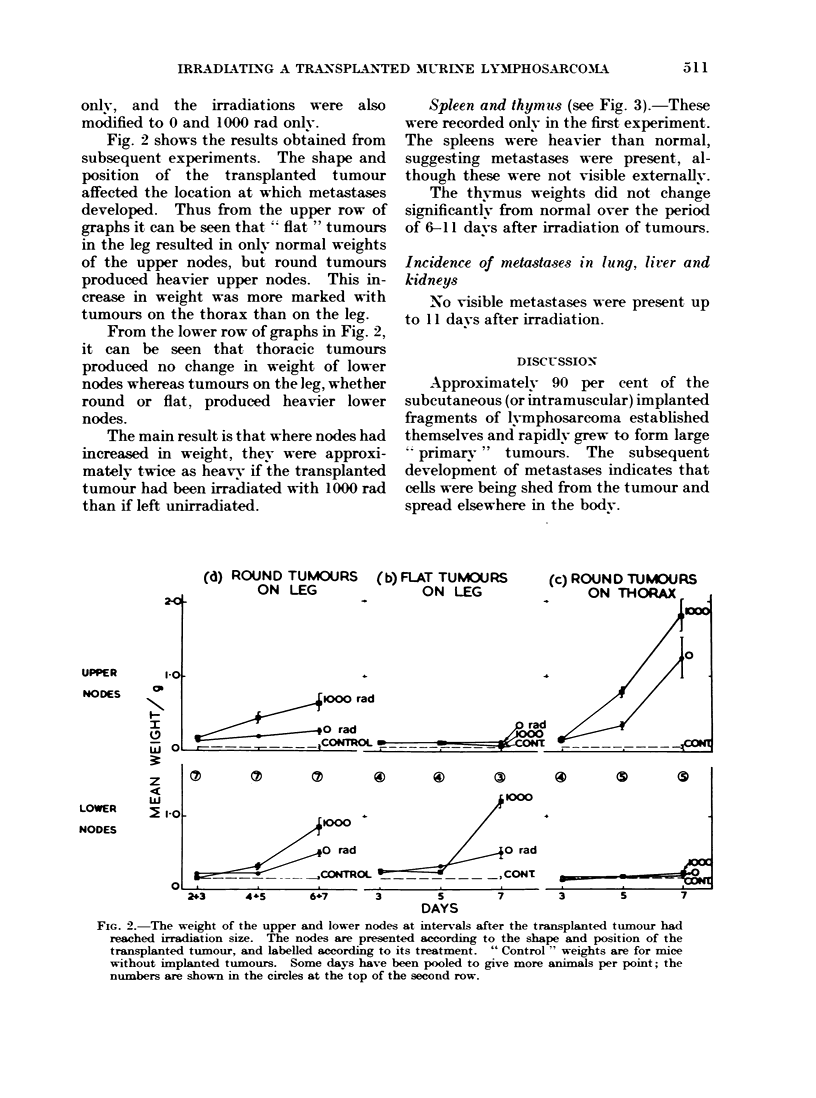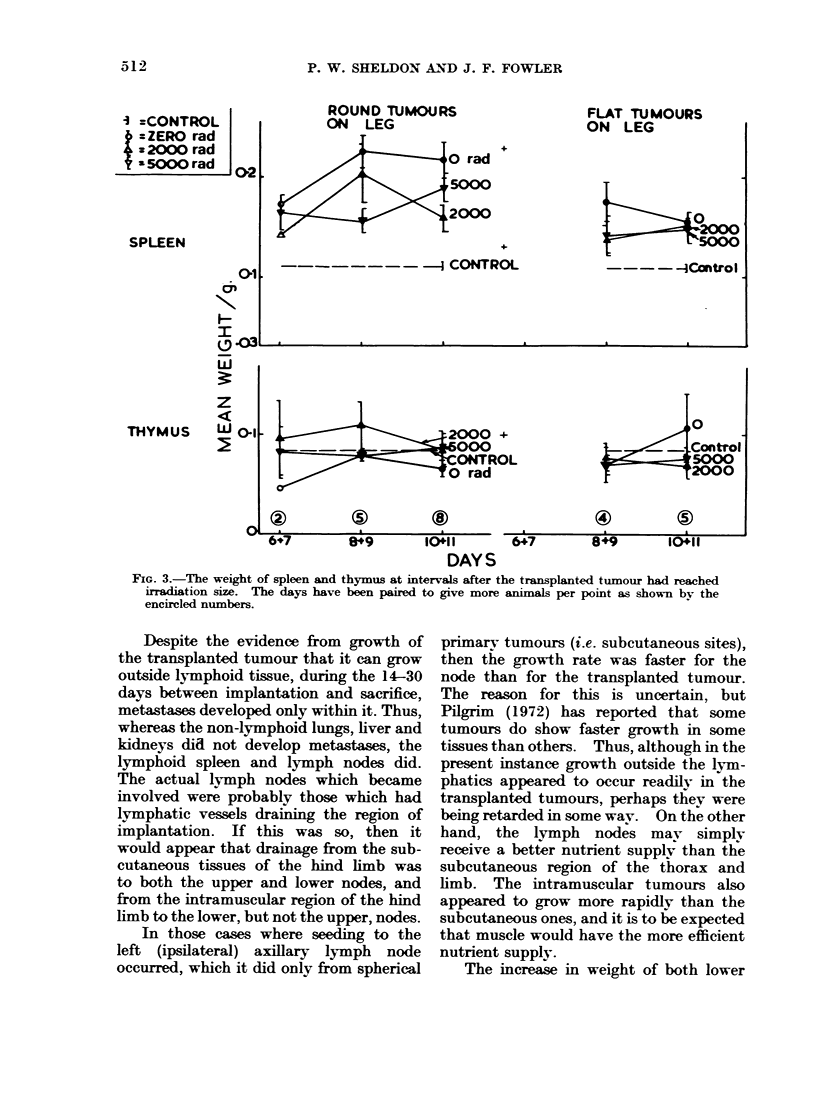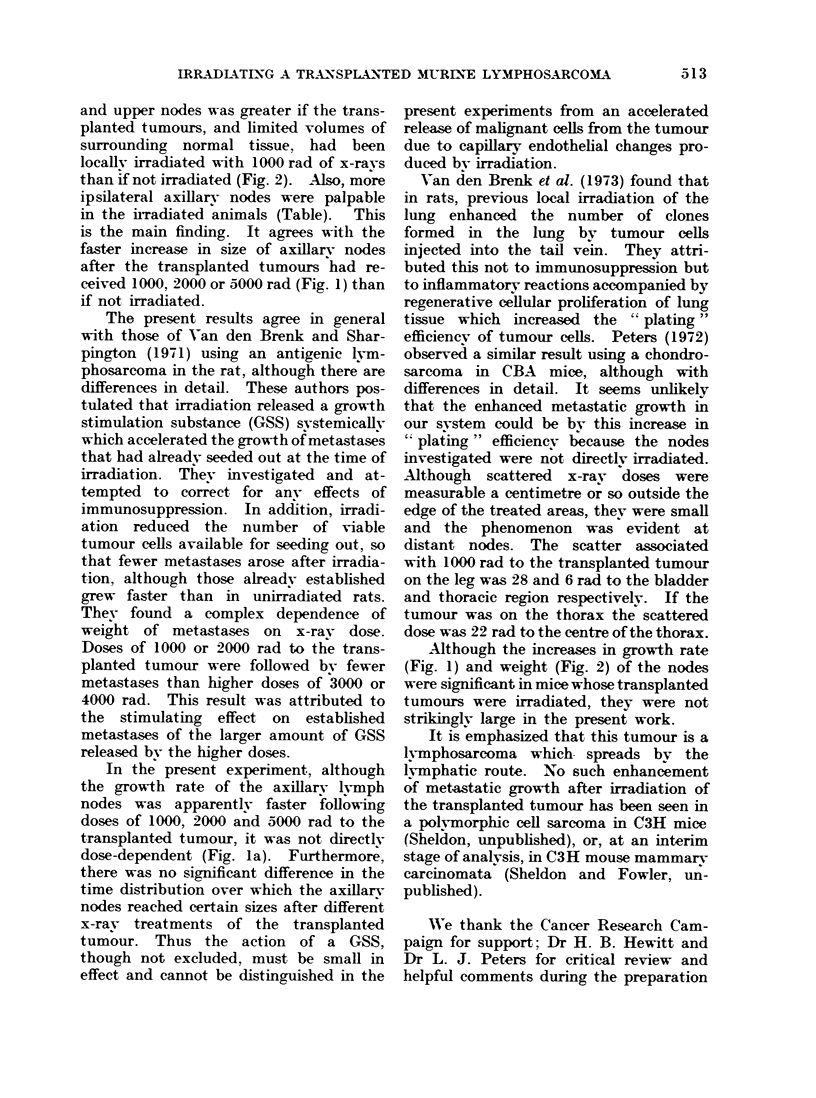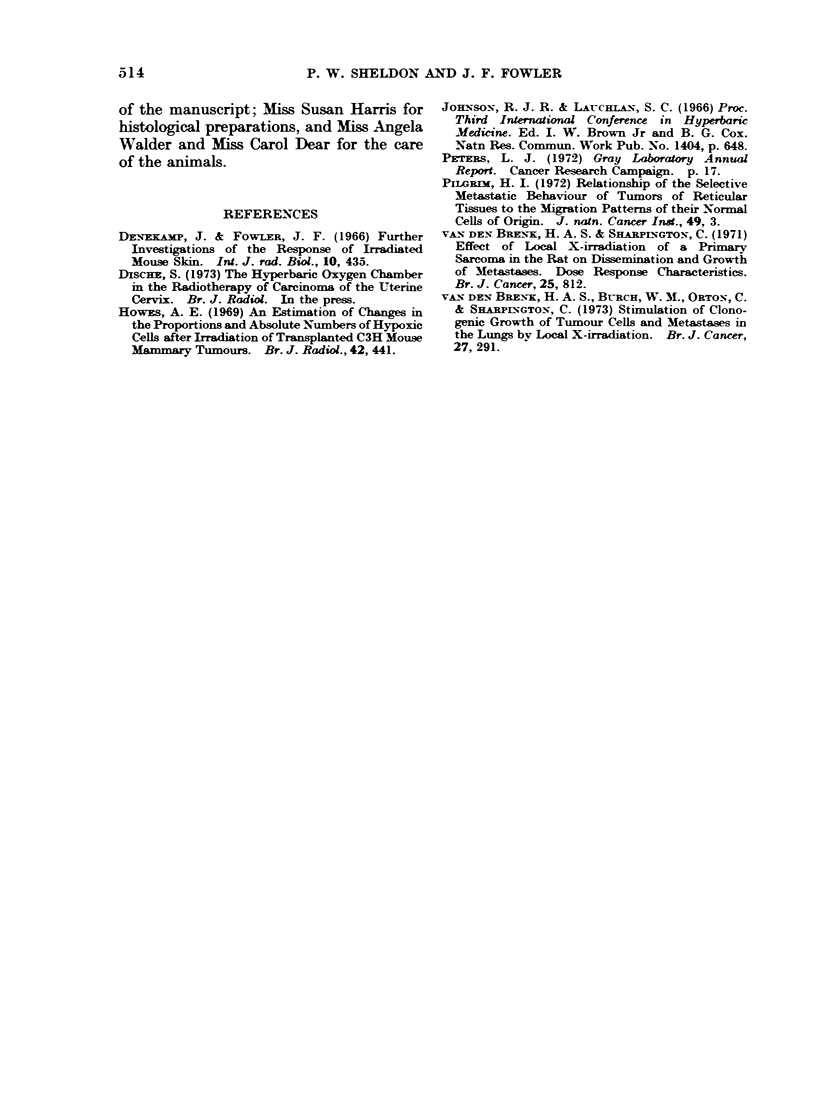# The Effect of Irradiating a Transplanted Murine Lymphosarcoma on the Subsequent Development of Metastases

**DOI:** 10.1038/bjc.1973.180

**Published:** 1973-12

**Authors:** P. W. Sheldon, J. F. Fowler

## Abstract

Mice were implanted with a lymphosarcoma on either the thorax or leg. Some of these tumours were given a single dose of 240 kV x-rays on reaching a predetermined size, whilst others were left unirradiated.

Metastases developed in specific lymph nodes, depending on the position of the transplanted tumour, and were larger if the tumour had been irradiated than if not. The growth rate of metastases in the ipsilateral axillary lymph node was faster than that of the transplanted tumours, irrespective of the radiation dose the tumour had received.


*Br. J. Cancer* (1973) **28**, 508

### THE EFFECT OF IRRADIATING A TRANSPLANTED MURINE LYMPHOSARCOMA ON THE SUBSEQUENT DEVELOPMENT OF METASTASES

P. W. SHELDON AND J. F. FOWLER

*From the Gray Laboratory, Mount Vernon Hospital, Northwood, Middlesex, HA6 2RN*

Received 16 July 1973. Accepted 9 August 1973

**Summary.—Mice were implanted with a lymphosarcoma on either the thorax or leg. Some of these tumours were given a single dose of 240 kV x-rays on reaching a predetermined size, whilst others were left unirradiated.**

**Metastases developed in specific lymph nodes, depending on the position of the transplanted tumour, and were larger if the tumour had been irradiated than if not. The growth rate of metastases in the ipsilateral axillary lymph node was faster than that of the transplanted tumours, irrespective of the radiation dose the tumour had received.**

In a tumour regrowth experiment performed at our laboratories in 1971 it was observed that those mice which had their transplanted tumour irradiated survived a shorter time than the control mice whose tumours had not been irradiated. Death was due to very large secondaries in the axillary lymph nodes in 13 of the 16 irradiated mice but in only 3 of the 13 unirradiated control mice.

This suggested that the irradiation was in some way promoting metastatic development, a possibility which appears to have been the subject of little research. However, this phenomenon was reported in man in 1966: radiotherapy in hyperbaric oxygen which induced rapid local regression of a primary tumour was apparently accompanied by an increase in growth rate of metastases outside the irradiated field (Johnson and Lauchlan, 1966). A follow-up involving more patients has, however, failed to substantiate this finding (R. J. Walton, personal communication to S. Dische, 1973).

The lack of experimental investigation is probably due to the difficulty of such a study, for unless the transplanted tumour is treated the animal will usually not survive long enough for metastases to develop. An example of this problem occurred in our laboratories in 1968. The irradiation of transplanted mammary carcinomata in C3H mice was followed by a significant incidence of lung metastases 4 months later, but no comparison with mice bearing unirradiated tumours could be made since they would have died of the transplanted tumour long before this time (Howes, unpublished). That investigation also revealed another typical problem: those mice which received a curative x-ray dose to the transplanted tumour had an incidence of metastases of only 3·6 per cent compared with 25·3 per cent for those receiving a non-curative dose; but was this a radiation enhancement or simply the result of prolonged seeding-time from a recurring transplanted tumour?

These problems were largely overcome by Van den Brenk and Sharpington (1971) who selected a rapidly metastasizing rat P-388 lymphosarcoma. They found a dose-dependent correlation between the irradiation of a primary tumour and the development of metastases.

Our tumour is also a rapidly metastasizing lymphosarcoma but of murine derivation.

### MATERIALS AND METHODS

The tumour, the lymphosarcoma P, was kindly supplied to us by Dr H. B. Hewitt. It originally arose spontaneously in a WHT/Ht mouse.

The tumour was cut into 1 mm cubes, and each cube was implanted subcutaneously into a 3-month old male syngeneic WHT/Ht mouse bred in the Gray Laboratory. The site of implantation was either centrally on the thorax (28 mice, as in the original tumour regrowth experiment) or else on the left hind limb (130 mice). In the latter case the tumours grew as 2 distinct types, being either spherical or diffuse. This appeared to be due to the difficulty of implanting subcutaneously in the leg because of the tight skin; hence, if the implant had been truly subcutaneous it grew as a sphere but if it had been intramuscular it grew diffusely along the muscle. Certainly over the thorax, where the skin was loose, all implants developed spherically.

The tumours were measured daily using calipers and the geometric mean diameters calculated. When these reached 6·5–7·5 mm (10–24 days after implantation) the mice were allocated into groups of 2 or 3, one mouse being left as an unirradiated control and the other one or 2 having their tumour irradiated. All mice in a group were subsequently sacrificed together. The data points consisted of several such groups sacrificed at different times.

Mice were anaesthetized for both implantation and irradiation with 60 mg/kg pentobarbitone sodium and subsequently revived with 0·5 mg per mouse of bemegride.

The x-irradiations were performed at 240 kV and 15 mA using a $\frac{1}{4}$ mm Cu + 1 mm Al filter to give a half value layer of 1·3 mm Cu.

Tumours implanted over the thorax were locally irradiated as described by Howes (1969) except that the system was modified to enable 4 mice to be irradiated simultaneously at a rate of 240 rad min$^{-1}$. Tumours implanted in the hind limb were irradiated as described by Denekamp and Fowler (1966) except that more of the leg was exposed to the beam (*i.e.* up to and including the inguinal lymph node). This restricted the number of mice that could be irradiated simultaneously to 4, the dose rate being 300 rad min$^{-1}$. Unirradiated tumour control mice were sham irradiated.

The parameters studied were: (1) the growth rate from caliper measurements of the transplanted tumour and of any metastasis in ipsilateral axillary lymph nodes; (2) the change in weight of certain lymph nodes and organs, including the upper nodes (consisting of the axillary and brachial lymph nodes), the lower nodes (consisting of the lumbar, renal, mesenteric and contralateral inguinal nodes), the spleen and the thymus. Several lymph nodes which were significantly heavier than normal were examined histologically and found to contain little cell debris but many apparently viable tumour cells. The viability of these cells was further suggested by the development of tumours after implantation of fragments of these nodes. These nodes and organs were also weighed in 10 normal control animals which had been exposed to neither a transplanted tumour nor irradiation; (3) the incidence of metastases in the lung, liver or kidneys.

### RESULTS

*Growth rate from caliper measurements* (see Fig. 1 and Table I)

The volume doubling time from 8 to 10 mm mean diameter was about 2 days for round transplanted tumours, whether in the leg or thorax, but only one day for metastases seeded to the left (ipsilateral) axillary lymph nodes, which also grew spherically. However, the flat transplanted tumours in the leg, which were more difficult to measure accurately, had a doubling time of only 1 day. They did not produce palpable lymph nodes.

*Change in node or organ weights*

*Upper nodes and lower nodes.*—In the first experiment transplanted tumours in the left leg were irradiated with either 0, 2000 or 5000 rad and the lymph nodes weighed on sacrificing the animals from 6 to 11 days later. It was found that the lower nodes had sometimes been subject to direct tumour infiltration, either from the transplanted tumour or from other involved nodes, and so the weights are not reproduced here. As this confluence was observed only after 8 days following implantation, subsequent experiments were performed at intervals of up to 7 days

FIG. 1.—A comparison of the transplanted " primary " tumour growth rate (upper row) with that of palpable left axillary lymph nodes (lower row)—all matched for size at the time of irradiation (Day 0). The transplanted tumours have been classified (a), (b) or (c) according to shape and position (see text). The palpable lymph nodes are displayed beneath the tumours from which they seeded. The dotted lines on the lymph node graphs represent the growth rates of the transplanted tumours. Open symbols represent the first experiment and closed symbols subsequent experiments. Standard errors of the mean are shown, but for clarity only alternate s.e. means are shown and only then if they exceed the symbol size. The number of mice per point is shown in the Table.

TABLE.—*The Number of Mice per Point in Fig. 1*

| | Transplanted tumour | | | | | | | | | | | | Left axillary lymph node from | | | | | | |
|---|---|---|---|---|---|---|---|---|---|---|---|---|---|---|---|---|---|---|---|
| Shape (site) | Round (leg) | | | | | Flat (leg) | | | | | Round (thorax) | | Round (leg) | | | | | Round (thorax) | |
| Dose (rad) to transplant (days) | 0* | 2000 | 5000 | 0 | 1000 | 0* | 2000 | 5000 | 0 | 1000 | 0 | 1000 | 0* | 2000 | 5000 | 0 | 1000 | 0 | 1000 |
| −7 | 1 | 4 | 3 | | | | | | | | 1 | 6 | | | | | | | |
| −6 | 3 | 5 | 6 | | | | | | | | 2 | 6 | | | | | | | |
| −5 | 6 | 6 | 7 | 8 | 2 | | | | 2 | | 7 | 6 | | 2 | | | | | |
| −4 | 7 | 11 | 10 | 8 | 10 | 1 | | | 4 | 3 | 11 | 9 | 3 | 3 | | | | | |
| −3 | 9 | 12 | 11 | 10 | 12 | 2 | 1 | 3 | 5 | 6 | 12 | 11 | 6 | 8 | 5 | 1 | 3 | 1 | 2 |
| −2 | 9 | 13 | 13 | 19 | 19 | 3 | 3 | 4 | 7 | 9 | 14 | 14 | 7 | 12 | 7 | 5 | 9 | 3 | 3 |
| −1 | 12 | 14 | 14 | 19 | 20 | 7 | 6 | 8 | 10 | 10 | 14 | 14 | 8 | 12 | 8 | 6 | 11 | 3 | 4 |
| 0 | 13 | 14 | 14 | 21 | 21 | 8 | 8 | 9 | 11 | 11 | 14 | 14 | 8 | 14 | 10 | 2 | 10 | 3 | 4 |
| 1 | 13 | 14 | 14 | 21 | 21 | 8 | 8 | 9 | 11 | 11 | 14 | 14 | 8 | 12 | 11 | | 8 | 2 | 5 |
| 2 | 13 | 14 | 14 | 21 | 21 | 8 | 8 | 9 | 11 | 11 | 14 | 14 | 7 | 11 | 9 | | 6 | 2 | 4 |
| 3 | 13 | 14 | 14 | 18 | 18 | 8 | 8 | 9 | 11 | 11 | 14 | 14 | 5 | 7 | 8 | | 2 | 2 | 3 |
| 4 | 13 | 14 | 14 | 14 | 14 | 8 | 8 | 9 | 7 | 7 | 10 | 10 | 3 | 4 | 7 | | 1 | 2 | 3 |
| 5 | 13 | 14 | 14 | 11 | 11 | 8 | 8 | 9 | 7 | 7 | 10 | 10 | 3 | 2 | 2 | | | | 2 |
| 6 | 13 | 14 | 14 | 7 | 7 | 8 | 8 | 9 | 3 | 3 | 5 | 5 | 1 | 2 | | | | | |
| 7 | 13 | 13 | 13 | 4 | 4 | 8 | 8 | 9 | 3 | 3 | 5 | 5 | 1 | 1 | | | | | |
| 8 | 12 | 12 | 12 | | | 8 | 8 | 9 | | | | | | | | | | | |
| 9 | 10 | 9 | 9 | | | 6 | 4 | 7 | | | | | | | | | | | |
| 10 | 8 | 7 | 7 | | | 4 | 4 | 5 | | | | | | | | | | | |
| 11 | | | | | | | 2 | 3 | | | | | | | | | | | |

* 0 represents the unirradiated mice in the first experiment.

The shape and site of the transplanted tumours, together with the x-ray doses given locally to them, are displayed in the headings of the first 3 data columns.

The 2 right-hand columns present data for the left axillary lymph nodes according to the treatment of the transplanted tumour from which they seeded.

only, and the irradiations were also modified to 0 and 1000 rad only.

Fig. 2 shows the results obtained from subsequent experiments. The shape and position of the transplanted tumour affected the location at which metastases developed. Thus from the upper row of graphs it can be seen that " flat " tumours in the leg resulted in only normal weights of the upper nodes, but round tumours produced heavier upper nodes. This increase in weight was more marked with tumours on the thorax than on the leg.

From the lower row of graphs in Fig. 2, it can be seen that thoracic tumours produced no change in weight of lower nodes whereas tumours on the leg, whether round or flat, produced heavier lower nodes.

The main result is that where nodes had increased in weight, they were approximately twice as heavy if the transplanted tumour had been irradiated with 1000 rad than if left unirradiated.

*Spleen and thymus* (see Fig. 3).—These were recorded only in the first experiment. The spleens were heavier than normal, suggesting metastases were present, although these were not visible externally.

The thymus weights did not change significantly from normal over the period of 6–11 days after irradiation of tumours.

*Incidence of metastases in lung, liver and kidneys*

No visible metastases were present up to 11 days after irradiation.

DISCUSSION

Approximately 90 per cent of the subcutaneous (or intramuscular) implanted fragments of lymphosarcoma established themselves and rapidly grew to form large " primary " tumours. The subsequent development of metastases indicates that cells were being shed from the tumour and spread elsewhere in the body.

FIG. 2.—The weight of the upper and lower nodes at intervals after the transplanted tumour had reached irradiation size. The nodes are presented according to the shape and position of the transplanted tumour, and labelled according to its treatment. " Control " weights are for mice without implanted tumours. Some days have been pooled to give more animals per point; the numbers are shown in the circles at the top of the second row.

FIG. 3.—The weight of spleen and thymus at intervals after the transplanted tumour had reached irradiation size. The days have been paired to give more animals per point as shown by the encircled numbers.

Despite the evidence from growth of the transplanted tumour that it can grow outside lymphoid tissue, during the 14–30 days between implantation and sacrifice, metastases developed only within it. Thus, whereas the non-lymphoid lungs, liver and kidneys did not develop metastases, the lymphoid spleen and lymph nodes did. The actual lymph nodes which became involved were probably those which had lymphatic vessels draining the region of implantation. If this was so, then it would appear that drainage from the subcutaneous tissues of the hind limb was to both the upper and lower nodes, and from the intramuscular region of the hind limb to the lower, but not the upper, nodes.

In those cases where seeding to the left (ipsilateral) axillary lymph node occurred, which it did only from spherical primary tumours (*i.e.* subcutaneous sites), then the growth rate was faster for the node than for the transplanted tumour. The reason for this is uncertain, but Pilgrim (1972) has reported that some tumours do show faster growth in some tissues than others. Thus, although in the present instance growth outside the lymphatics appeared to occur readily in the transplanted tumours, perhaps they were being retarded in some way. On the other hand, the lymph nodes may simply receive a better nutrient supply than the subcutaneous region of the thorax and limb. The intramuscular tumours also appeared to grow more rapidly than the subcutaneous ones, and it is to be expected that muscle would have the more efficient nutrient supply.

The increase in weight of both lower and upper nodes was greater if the transplanted tumours, and limited volumes of surrounding normal tissue, had been locally irradiated with 1000 rad of x-rays than if not irradiated (Fig. 2). Also, more ipsilateral axillary nodes were palpable in the irradiated animals (Table). This is the main finding. It agrees with the faster increase in size of axillary nodes after the transplanted tumours had received 1000, 2000 or 5000 rad (Fig. 1) than if not irradiated.

The present results agree in general with those of Van den Brenk and Sharpington (1971) using an antigenic lymphosarcoma in the rat, although there are differences in detail. These authors postulated that irradiation released a growth stimulation substance (GSS) systemically which accelerated the growth of metastases that had already seeded out at the time of irradiation. They investigated and attempted to correct for any effects of immunosuppression. In addition, irradiation reduced the number of viable tumour cells available for seeding out, so that fewer metastases arose after irradiation, although those already established grew faster than in unirradiated rats. They found a complex dependence of weight of metastases on x-ray dose. Doses of 1000 or 2000 rad to the transplanted tumour were followed by fewer metastases than higher doses of 3000 or 4000 rad. This result was attributed to the stimulating effect on established metastases of the larger amount of GSS released by the higher doses.

In the present experiment, although the growth rate of the axillary lymph nodes was apparently faster following doses of 1000, 2000 and 5000 rad to the transplanted tumour, it was not directly dose-dependent (Fig. 1a). Furthermore, there was no significant difference in the time distribution over which the axillary nodes reached certain sizes after different x-ray treatments of the transplanted tumour. Thus the action of a GSS, though not excluded, must be small in effect and cannot be distinguished in the present experiments from an accelerated release of malignant cells from the tumour due to capillary endothelial changes produced by irradiation.

Van den Brenk *et al.* (1973) found that in rats, previous local irradiation of the lung enhanced the number of clones formed in the lung by tumour cells injected into the tail vein. They attributed this not to immunosuppression but to inflammatory reactions accompanied by regenerative cellular proliferation of lung tissue which increased the "plating" efficiency of tumour cells. Peters (1972) observed a similar result using a chondrosarcoma in CBA mice, although with differences in detail. It seems unlikely that the enhanced metastatic growth in our system could be by this increase in "plating" efficiency because the nodes investigated were not directly irradiated. Although scattered x-ray doses were measurable a centimetre or so outside the edge of the treated areas, they were small and the phenomenon was evident at distant nodes. The scatter associated with 1000 rad to the transplanted tumour on the leg was 28 and 6 rad to the bladder and thoracic region respectively. If the tumour was on the thorax the scattered dose was 22 rad to the centre of the thorax.

Although the increases in growth rate (Fig. 1) and weight (Fig. 2) of the nodes were significant in mice whose transplanted tumours were irradiated, they were not strikingly large in the present work.

It is emphasized that this tumour is a lymphosarcoma which spreads by the lymphatic route. No such enhancement of metastatic growth after irradiation of the transplanted tumour has been seen in a polymorphic cell sarcoma in C3H mice (Sheldon, unpublished), or, at an interim stage of analysis, in C3H mouse mammary carcinomata (Sheldon and Fowler, unpublished).

We thank the Cancer Research Campaign for support; Dr H. B. Hewitt and Dr L. J. Peters for critical review and helpful comments during the preparation of the manuscript; Miss Susan Harris for histological preparations, and Miss Angela Walder and Miss Carol Dear for the care of the animals.

### REFERENCES

DENEKAMP, J. & FOWLER, J. F. (1966) Further Investigations of the Response of Irradiated Mouse Skin. *Int. J. rad. Biol.*, **10**, 435.

DISCHE, S. (1973) The Hyperbaric Oxygen Chamber in the Radiotherapy of Carcinoma of the Uterine Cervix. *Br. J. Radiol.* In the press.

HOWES, A. E. (1969) An Estimation of Changes in the Proportions and Absolute Numbers of Hypoxic Cells after Irradiation of Transplanted C3H Mouse Mammary Tumours. *Br. J. Radiol.*, **42**, 441.

JOHNSON, R. J. R. & LAUCHLAN, S. C. (1966) *Proc. Third International Conference in Hyperbaric Medicine*. Ed. I. W. Brown Jr and B. G. Cox. Natn Res. Commun. Work Pub. No. 1404, p. 648.

PETERS, L. J. (1972) *Gray Laboratory Annual Report*. Cancer Research Campaign. p. 17.

PILGRIM, H. I. (1972) Relationship of the Selective Metastatic Behaviour of Tumors of Reticular Tissues to the Migration Patterns of their Normal Cells of Origin. *J. natn. Cancer Inst.*, **49**, 3.

VAN DEN BRENK, H. A. S. & SHARPINGTON, C. (1971) Effect of Local X-irradiation of a Primary Sarcoma in the Rat on Dissemination and Growth of Metastases. Dose Response Characteristics. *Br. J. Cancer*, **25**, 812.

VAN DEN BRENK, H. A. S., BURCH, W. M., ORTON, C. & SHARPINGTON, C. (1973) Stimulation of Clonogenic Growth of Tumour Cells and Metastases in the Lungs by Local X-irradiation. *Br. J. Cancer*, **27**, 291.